# Structural Insights in Multifunctional Papillomavirus Oncoproteins

**DOI:** 10.3390/v10010037

**Published:** 2018-01-15

**Authors:** Irina Suarez, Gilles Trave

**Affiliations:** Équipe Labellisée Ligue 2015, Department of Integrated Structural Biology, Institut de Génétique et de Biologie Moléculaire et Cellulaire (IGBMC), INSERM U1258/CNRS UMR 7104/Université de Strasbourg, 1 rue Laurent Fries, BP 10142, F-67404 Illkirch, France; suarezi@igbmc.fr

**Keywords:** papillomaviruses, oncoproteins, structure, X-ray, NMR, virus-host interactomics

## Abstract

Since their discovery in the mid-eighties, the main papillomavirus oncoproteins E6 and E7 have been recalcitrant to high-resolution structure analysis. However, in the last decade a wealth of three-dimensional information has been gained on both proteins whether free or complexed to host target proteins. Here, we first summarize the diverse activities of these small multifunctional oncoproteins. Next, we review the available structural data and the new insights they provide about the evolution of E6 and E7, their multiple interactions and their functional variability across human papillomavirus (HPV) species.

## 1. Introduction

Papillomaviruses (PVs) constitute a large family of small oncogenic DNA viruses that infect mucosal or cutaneous epithelia [1,2]. PVs have been found in most vertebrate species investigated including human [3], other mammals, birds, reptiles [4], and very recently in fish [5,6]. PVs contain a very small double-stranded circular DNA genome of 7000 to 8000 base pairs [3]. Papillomaviruses are classified into distinct genera, species, types, or subtypes whenever the DNA sequences of their L1 gene share less than 60%, 70%, 90% or 98% identity, respectively. To date, 353 PV types have been identified and classified into 49 distinct genera [7,8] (see curated list at Papillomavirus Episteme (PaVE); https://pave.niaid.nih.gov/#home) [7]. They include more than 200 HPV types belonging to five distinct genera (alpha, beta, gamma, mu, and nu). Most alpha HPVs display a mucosal tropism while most beta, mu and nu HPVs infect the skin. However, HPV species alpha-2, alpha-4, and alpha-8 are found in skin warts [9] whereas HPV species beta-3 seems to be more commonly found in the nasal cavity than in skin [10], and therefore may display a mucosal tropism [11]. Gamma HPV appear to display both cutaneous and mucosal tropism [9].

All PVs induce proliferation of the infected cells. This proliferation mobilizes the cellular DNA replication and protein production machinery, which are hijacked by the virus to the benefit of its own replication [1,2,3,12]. In general, proliferation lasts only for a short time, allowing the production of virions ready to infect another individual. This activates an immunological response from the host, leading in most cases to virus clearance. Most HPV types that are designated as “low-risk” generate mild benign pathogenic effects, such as skin warts, mucosal lesions, or in the worst case, mucosal condylomas that require surgery [1,2,3]. However, for a subset of HPV types dubbed “high-risk”, viral genes sometimes do not get fully eradicated by the host. All or part of the viral genome remains maintained in at least one host cell, either in an episomal form or inserted in the host cell genome [13]. These remaining viral oncogenes have the capacity to promote, over a long period that may last up to three decades, further changes to the infected cell, which may eventually lead to cancer [1,2,3,12,14]. Indeed, high-risk mucosal HPVs are responsible for practically 100% of cervical, anal, rectal, and penile cancers, as well as for an increasingly high proportion of oropharyngeal cancers [15].

The two main viral HPV oncogenes required to establish and maintain the tumorigenic phenotype encode two early expressed oncoproteins, called E6 and E7. Both E6 and E7 are very small proteins (in most mammalian PVs about 150 and 100 amino acids, respectively). Nonetheless, they are responsible for most of the proliferative and transforming events that lead to carcinogenesis [12,16,17,18]. Altogether, E6 and E7 display pro-proliferative and anti-apoptotic effects as well as cell adhesion, cell polarity, and cell differentiation-altering properties that facilitate a transient period of proliferation of the infected epithelial cells.

Remarkably, turning off the expression of E6 oncoprotein in HPV-positive cancer-derived cells by means of RNA interference induces growth arrest followed by either apoptosis or senescence [1,19,20,21]. This indicates that HPV-positive cancers are “addicted” to E6 expression for their survival, so that inhibition of key E6 functions may represent a promising strategy for counteracting the growth of HPV-positive tumors. Indeed, blocking E6 molecular activities by means of E6-binding recombinant proteins [22], peptides [23,24,25], or antibodies [26,27] has been shown to drive specifically growth arrest and/or death of HPV-positive tumor cells. These data indicate that well-designed small molecule ligands of E6 will represent promising avenues for therapy of HPV-positive cancers. Yet, a prerequisite for the ab initio design of such small molecule inhibitors, and for the rationale improvement of current low-affinity E6 inhibitors [28,29,30], is to obtain high-resolution information on the three-dimensional structure of E6 proteins alone or in complex with their cellular targets.

## 2. E6 and E7 Are Multifunctional Proteins

### 2.1. E6 and E7 Interact with Large Numbers of Host Target Proteins

For the best-studied mammalian PVs, both E6 and E7 have been found to interact with numerous distinct cellular target proteins involved in a variety of cellular functions [16,17,31,32,33,34,35,36]. Importantly, E6 proteins from distinct HPV species recognize distinct subsets of the full panel of potential E6 targets [31]. This exquisite capacity of different E6 proteins to recognize particular pools of targets likely contributes to the particular biological traits of each HPV type in terms of tropism, viral cycle, or pathogenicity. The same multifunctional character, undergoing variations across HPV species, has been observed for HPV E7 oncoproteins [31].

### 2.2. Viral Domain-Motif Hijacking Strategies Explain E6 and E7 Multifunctionality

The ability of the E6 and E7 proteins to recruit large numbers of different and often functionally unrelated proteins is at first thought surprising, since such small proteins cannot, in principle, present many distinct interaction surfaces. This can, however, be explained by considering the “domain-motif hijacking strategies” employed by many viral or bacterial pathogen proteins to disrupt or reprogram particular functions of the infected hosts [37]. In brief, a large part of cellular protein–protein interactions boil down to specific interactions between folded globular domains and short intrinsically unfolded linear motifs, which get folded upon binding to their target domains [38]. Viral and bacterial infectious agents produce proteins bearing the ability to divert these “domain-motif functional interaction networks” for the sake of their own lifecycle [37,39].

One can cite at least three well-documented examples of domain-motif network hijacking by the viral oncoproteins E6 and E7.

The E7 proteins from most papillomaviruses contain a conserved LxCxE motif, also found in many host proteins. The LxCxE motif is specialized in the recognition of the “pocket domain” of members of the retinoblastoma (Rb) protein family, including the Retinoblastoma tumor suppressor pRB (RB1), p107 (RBL1), and p130 (RB2) [1,17]. The binding of E7 via its LxCxE motif to Rb pocket proteins, and their subsequent proteasomal degradation (as discussed later in this review) disrupts complexes between Rb proteins and E2 transcription factors (E2F). Consequently, the active E2F factors released in this way facilitate cell division and its subsequent hijacking by the virus.

All E6 oncoproteins from High-Risk Mucosal HPVs (hrm-HPVs) present a conserved C-terminal PDZ-Binding Motif (PBM) that allows them to bind, and sometimes provoke the cellular degradation of, multidomain proteins containing PDZ domains. PDZ domains, named from the first letters of three proteins sharing such domains (post synaptic density protein (PSD95), Drosophila disc large tumor suppressor (Dlg1), and zonula occludens-1 protein (zo-1)) are involved in various processes including cell polarity, cell adhesion, and apoptosis [40,41,42,43,44,45,46].

In addition, most E6 proteins from mammalian papillomaviruses contain a charged hydrophobic pocket, which recognizes peptides prone to alpha helical structure that presents a conserved LxxLL consensus sequence interspaced with acidic residues [16,47,48,49,50]. This pocket allows E6 proteins to recruit a variety of host cellular proteins containing LxxLL motifs and involved in a variety of apparently unrelated functions, such as the ubiquitin ligase E6-Associated Protein (E6AP) [47], the focal adhesion protein paxillin [49], the cell fate-determining Mastermind-like transcriptional coactivator 1 (MAML1) [51,52,53], or the antiviral Interferon Regulatory transcriptional Factor 3 (IRF3) [54]. In all these proteins, the LxxLL motif is found in a region predicted as intrinsically unfolded, ensuring the accessibility of the motif for interaction with E6.

### 2.3. E6 and E7 Divert the Host Ubiquitination Machinery

Frequently, E6 and E7 not only bind to their targets but also provoke their accelerated destruction by the Ubiquitin Proteasome System (UPS) [55,56]. This is generally achieved via a tripartite interaction, in which the viral oncoprotein recruits, on the one hand, a UPS enzyme (generally, an E3 ubiquitin ligase) and on the other hand a target cellular protein. The target protein is subsequently poly-ubiquitinated, then degraded by the proteasome system, while the viral oncoprotein and the ubiquitin ligase are recycled for degrading the next molecule of target protein. The best documented case of such “UPS hijacking” by HPVs is performed by the E6 oncoprotein of high-risk mucosal (hrm) HPVs. The Hrm-HPV E6 binds to E6AP (also called UbE3A), a cellular E3 ubiquitin ligase containing a HECT (Homologous to E6AP C-Terminal) domain specialized in poly-ubiquitination of target proteins. The resulting E6/E6AP complex then recruits the p53 anti-apoptotic tumor suppressor protein, provoking its poly-ubiquitination and subsequent degradation by the proteasome [57,58]. In a somehow comparable process, the E7 oncoprotein of hrm-HPVs binds to the cullin 2 ubiquitin ligase, resulting in the proteasome-dependent degradation of pRb [59,60]. In a recent work, screening of a library expressing 590 proteins related to the UPS confirmed E6AP and members of cullin family as UPS targets of hrm HPV E6 and E7, respectively, and identified novel potential targets for both oncoproteins [61].

### 2.4. E6 and E7 and Nucleic Acids

While the best studied molecular activities of E6 and E7 proteins are related to their ability to interact with target cellular proteins, both E6 and E7 have early been suggested to interact also with nucleic acids [62,63,64]. This hypothesis was based on the fact that both E6 and E7 contain conserved zinc-binding domains with sequences distantly reminiscent of the zinc finger domains of transcription factors, including repeats of four conserved cysteine residues. Indeed, a subset of E6 proteins from high-risk mucosal HPV types was found to interact with high affinity and selectivity with 4-way DNA junctions [65] and the residues responsible for DNA binding were localized within the C-terminal zinc-binding domain of these E6 proteins [66,67,68]. The E6 proteins of high-risk mucosal HPVs have also been found to be RNA-binding proteins that can inhibit splicing of pre-mRNAs [69]. How these nucleic-acid binding properties are utilized during the virus lifecycle, and whether they contribute to the oncogenic phenotype, is still poorly understood.

### 2.5. The Multifunctionality Issue: How to Make Sense of Complexity?

The multifunctional character of PV oncoproteins raises the problem of how to rank/evaluate the individual contributions of their numerous interactions and of the subsequent biochemical reactions to viral tropism, lifecycle, and pathogenesis. At the limits, one may either consider that all observed interactions are equally and indistinctively relevant (“big-bag complexity” view point), or consider on the contrary that only a few interactions are relevant with all the rest being artefactual or fortuitous (“extreme reductionism” view point). A way to reconcile these opposite view points may be to consider that each identified interaction of E6 or E7 participates in viral tropism, lifecycle, and pathogenesis according to a given “weight”, and that the observed phenotype emerges from the combination of all the differently weighted interactions and subsequent reactions. In such a “combinatorial-weighted” approach, the list of interactions taken into consideration remains the same for each E6 or E7 protein across the HPV phylogenetic tree, but the weight allocated to each individual interaction varies for each viral type considered.

How could one quantify the weight allocated to each viral-host interaction? One approach may be to use quantitative information derived from mass spectrometry (MS). In a remarkable work, White and collaborators [31,36] have used E6 proteins from 16 different HPV types belonging to eight different species and two different HPV genera (α and β), to identify 153 E6-binding cellular proteins. Each E6 protein considered was found to bind detectably to only a reduced subset of these 153 proteins. In addition, each E6 protein varied in its efficiency of pulling down each particular partner. This information was quantified in the form of a “Normalized Weighted D-score” that computed the uniqueness, abundance, and reproducibility of each identified E6-target interaction. In another study by Thomas et al. [70], resin beads pre-saturated with synthetic PDZ-Binding Motifs (PBMs) derived from 10 different HPV E6 proteins were incubated with keratinocyte extracts, leading to the pull-down and MS-based identification of 19 E6-binding PDZ domain-containing host cell proteins. Thanks to a reproducible protocol where only the PBM sequence varied, the normalized mean numbers of peptides of each pulled-down host protein identified by MS provided quantitative scores representative of the preferences of each E6 protein.

Another approach to allocating a quantitative weight to the different viral-host interactions could be to measure the affinity displayed by the viral protein towards each potential cellular partner. Recently, Vincentelli et al. [71] bacterially expressed 209 PDZ domains, representing 79% of the entire complement of human PDZ domains (the “PDZome”) and used a high-throughput chromatographic approach (the holdup assay) to systematically measure in vitro the affinities of each expressed PDZ domain towards the PBMs of E6 proteins from the two highest-risk mucosal HPV types, 16 and 18. The data were represented in the form of “PDZome-binding specificity profiles”, which allow visualizing and comparing at one glance the binding strengths of each E6 PBM towards all individual domains of the human PDZome.

## 3. Sequence and Structure of E6 and E7 Oncoproteins

### 3.1. Amino Acid Sequence Features of E6 and E7

The E6 and E7 sequences are present in the large majority of vertebrates (mammalian, avian, and reptilian) papillomaviruses characterized to date [6,7]. However, there are exceptions, including a few human papillomaviruses, in which the E6 sequences are missing [72,73]. The recently discovered fish papillomaviruses are devoid of both E6 and E7 sequences [6].

**Figure 1:**
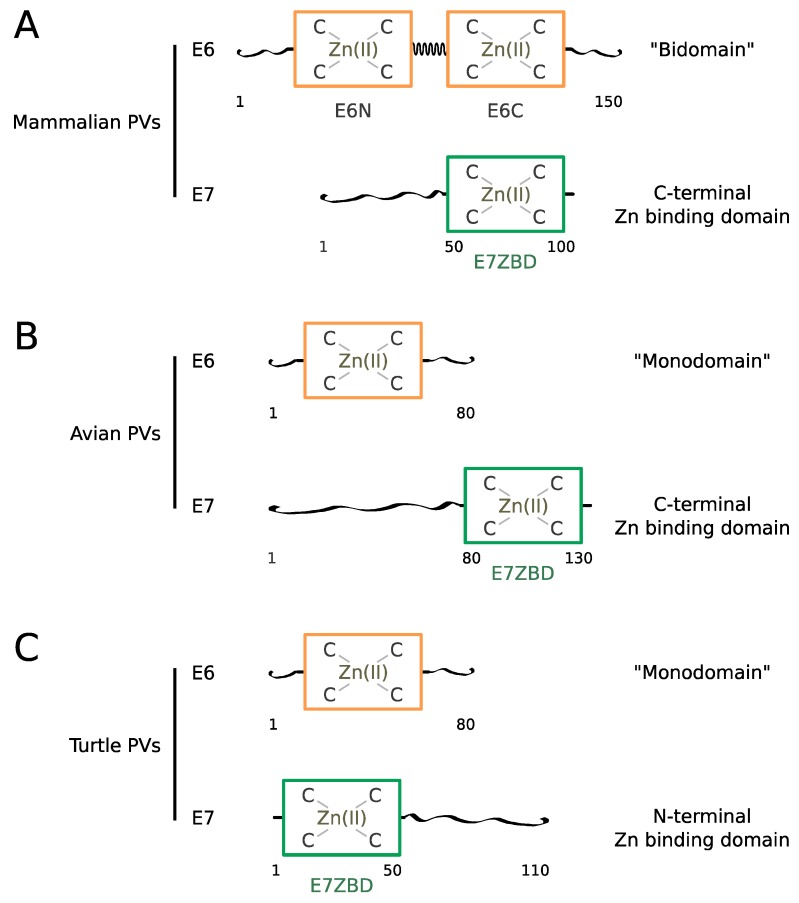
Schematics of E6 and E7 sequences across distinct vertebrates: (**A**) mammals; (**B**) birds; (**C**) turtles. Folded zinc-binding domains and unfolded regions are represented as rectangles and extended strings, respectively. Approximate amino-acid numbering is indicated below the schemes.

In most mammalian PVs, the E6 sequence spans about 150 amino acids comprising two repeated 70-residue zinc-binding domains called E6N and E6C [68,74], connected by a linker helix [75] (Figure 1A). The tandem repeat is surrounded by N-terminal and C-terminal extensions displaying higher sequence variability and a higher propensity to intrinsic disorder.

Mammalian E7 proteins span about 100 residues comprising a 50 residue-long N-terminal disordered region [76,77] followed by a 50-residue-long C-terminal zinc-binding domain, which folds as an obligate homodimer [76,78] (Figure 1A). The E7 zinc-binding domain, sometimes called CR3 in former publications, will be referred to as “E7-ZBD” from now on in this text.

In avian papillomaviruses, the E6 protein is constituted by a single zinc-binding 80-residue domain whereas the E7 protein contains a presumably disordered N-terminal region encompassing about 80 residues followed by a folded C-terminal zinc-binding domain encompassing about 50 residues [6,7,79] (Figure 1B).

Turtle papillomaviruses [6,7,80] also comprise an 80-residue single-domain E6 protein and a 110-residue E7. Curiously, in the two turtle papillomaviruses identified, the predicted 50-residue E7 zinc-binding domain is situated at the N-terminus and the presumably disordered region at the C-terminus (Figure 1C), in contrast to what is observed in both mammalian and avian E7.

The presumably disordered regions, both in E6 and E7, harbor “mimics” of host short linear interaction motifs (sLiMs), allowing E6 and E7 to hijack cellular signaling networks involving such interaction motifs, as observed for many viral proteins [37,39]. This is the case of the PDZ-binding motif found at the C-terminus of E6 from high-risk mucosal (hrm) HPVs as well as the LxCxE Rb-binding motif found within the disordered region of most mammalian E7 proteins.

As will be discussed later in this review, structural data on two phylogenetically distant mammalian E6 proteins (from HPV16 and BPV1) [75] have shown that E6-LxxLL motif recognition is conserved across mammalian PVs, and that this property is related to the bi-domain structure of these E6 proteins. Since the E6 proteins of bird and turtle papillomavirus are constituted of a single domain, E6-LxxLL recognition is likely to be an acquired property that emerged for the mammalian papillomaviruses only.

### 3.2. History of Progress towards E6 and E7 Tri-Dimensional Structures

The E6 and E7 proteins were identified as major PV oncoproteins in the mid-80s [81,82,83,84,85], immediately promoting the first attempts to produce these proteins in recombinant form [86,87,88,89]. Nevertheless, the first structures of the isolated zinc-binding domains of either E6 [67] or E7 [76,78] were only released in 2006, whereas the first structures of full-length mammalian PV E6 proteins were released in 2013 [75], almost thirty years after the discovery of E6 and E7 oncoproteins. This was mainly due to difficulties in producing homogeneous soluble samples of recombinant E6 and E7 proteins. The search for soluble E6 samples amenable to structural analysis has been particularly arduous, going through the following steps: (i) experimental delimitation of the two zinc-binding domains [68,74,90,91]; (ii) solubilization of E6 by means of fusion to the highly soluble bacterial Maltose Binding Protein (MBP) [92,93,94,95]; (iii) biophysical characterization of E6 oligomers [92,93,94,96,97]; (iv) separation of soluble monomeric MBP-E6 fusions from soluble aggregated MBP-E6 fusions [92,93,94]; (v) solubilizing mutagenesis of E6 proteins to replace exposed non-conserved cysteine residues that promote intermolecular disulfide bridging during E6 purification and storage [92,93,94]; (vi) solubilizing mutagenesis of E6 proteins to replace surface exposed hydrophobic residues that promote oligomerization of purified E6 [91]; (vii) solubilization and stabilization of E6 proteins by binding to their cognate LxxLL motif [75,95,98]. Therefore, it was the interdependent progress in understanding the mechanisms of E6 self-association and in finding strategies to prevent these mechanisms that ultimately led to the resolution of structures of E6 proteins [67,75,91,98].

### 3.3. Structure of the Zinc-Binding Domains of E6

The folded regions of E6 and E7 contain zinc-binding folds that have not yet been observed elsewhere in the living kingdom, although some of their structural characteristics can be found within other proteins.

**Figure 2:**
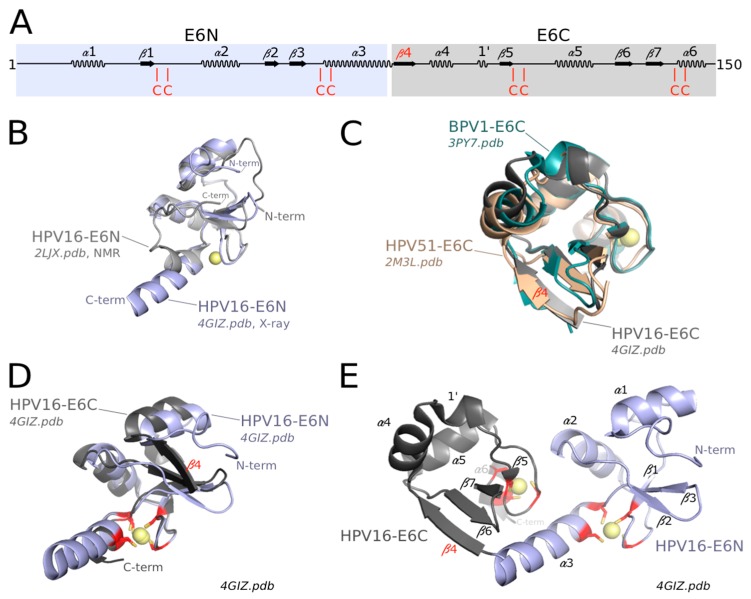
Architecture of mammalian human papillomavirus (HPV) E6. (**A**) Secondary structure elements of mammalian HPV16 E6. E6N is shaded in blue, E6C is shaded in gray; (**B**) Superimposition of HPV16 E6N domain solved by NMR [91] and HPV16 E6N domain from the crystal structure of the E6/E6AP LxxLL complex [75]; (**C**) Superimposition of HPV16 E6C domain from the crystal structure of the E6/E6AP LxxLL complex [75], HPV51 E6C domain solved by NMR [101] and bovine papillomavirus 1 (BPV1) E6C domain from the crystal structure of the BPV1 E6/paxillin LxxLL complex [75]; (**D**) Superimposition of HPV16 E6N and E6C domains from the crystal structure of the HPV16 E6/E6AP LxxLL complex [75]; (**E**) Structure of LxxLL-bound mammalian HPV16 E6 [75] (LxxLL motif not shown for clarity). Zinc-coordinating cysteines are highlighted in red. Secondary elements are numbered as in [75]. Spheres are Zinc (II) atoms. All structural views in this article were prepared using Pymol (http://www.pymol.org/).

Two structures of distinct mammalian PV E6N domains are available to date, one from HPV16 and another one from the phylogenetically distant bovine PV, bovine papillomavirus 1 (BPV1). The structure of HPV16 E6N has been solved both by Nuclear Magnetic Resonance (NMR) in the form of an isolated fragment of E6 [91] and by crystallography in the frame of LxxLL motif-bound full-length HPV16 E6 [75,98]. The solution structure of the isolated E6N domain and its crystal structure within the crystallized full-length E6 are well superimposed (Figure 2B). The structure of the BPV1 E6N domain, as observed in the crystal structure of the BPV1 E6/paxillin complex [75] is homologous to that of HPV16 E6N, except for an N-terminal region of BPV1 E6N which was not visible in the BPV1 E6 crystal. The core of the HPV16 E6N fold consists of a three-stranded β-sheet and three α-helices, reinforced by a peripheral zinc-binding site. Two zinc-liganding cysteines are contributed by a knuckle situated at the junction between strand β1 and helix α2, while the other two zinc-liganding cysteines are contributed by the C-terminal α-helix α3 (Figure 2A,D,E). The BPV1 E6N lacks the N-terminal α-helix, which may exist as a part of the nonobservable region in the crystal (See Supplementary Figure S3 of [75]). In HPV16 E6N, the first ten N-terminal residues adopt an extended and rather flexible structure, which is, however, anchored to the hydrophobic core by phenylalanine F2 [75,91]. This explains why mutagenesis of residue F2 was previously found to alter various E6 activities [99,100], despite the fact that F2 is not involved in the interfaces with E6AP and p53 in the E6/E6AP and E6/E6AP/p53 complexes [75,98]. The conserved C-terminal helix of E6N domains constitutes the start of the “linker helix” that connects the E6N and E6C domains within the crystal structures of HPV16 E6 and BPV1 E6 [75] (Figure 2E).

Three structures of distinct mammalian PV E6C domains are available to date: from HPV16 [75,91], from the related high-risk mucosal HPV 51 [101], and from BPV1 [75]. As observed for HPV16 E6N, HPV16 E6C displays identical structures when solved either as an isolated fragment by NMR or as part of full-length HPV16 E6 by crystallography [75]. Moreover, the HPV51 E6C (solved in isolation by NMR) [101] and the BPV1 E6C (as part of the crystal structure of full-length BPV1 E6 [75]) are perfectly superimposed on the structure of HPV16 E6C (Figure 2C).

Despite sharing only 10% sequence identity, the E6N and E6C domains have homologous structures, with the exception of the flanking N- and C-terminal regions (Figure 2A,D). Whereas the N-terminal region of HPV16 E6N is a flexible loop, the N-terminus of all solved E6C domains (from HPV16, HPV51 and BPV1) folds as an additional β-strand (β4), which extends the β-sheet. The C-terminal region of E6N corresponds to the start of the interdomain linker (see below), while the C-terminus of E6C harbors a PDZ-Binding Motif in high-risk mucosal HPVs and distinctive sequences in other types. The structural homology between E6N and E6C confirms sequence-based analyses [6,62], which suggests that they arose from duplication of a single-domain ancestor. This hypothesis is further supported by the fact that the E6 proteins of avian and turtle PVs are composed of a single zinc-binding domain. Indeed, the single-domain avian PV E6 [79] is similar, by its sequence and overall fold, to the E6C domains of mammalian PV E6.

Within full-length E6, the two domains E6N and E6C are connected by a linker. In the crystal structures of full-length E6 bound to LxxLL motifs [75], this linker forms a helix, in continuation of helix α3 at the exit of the E6N zinc binding site (Figure 2E).

### 3.4. Structure of the Zinc-Binding Domains of E7 and Comparison to E6 Domains

**Figure 3:**
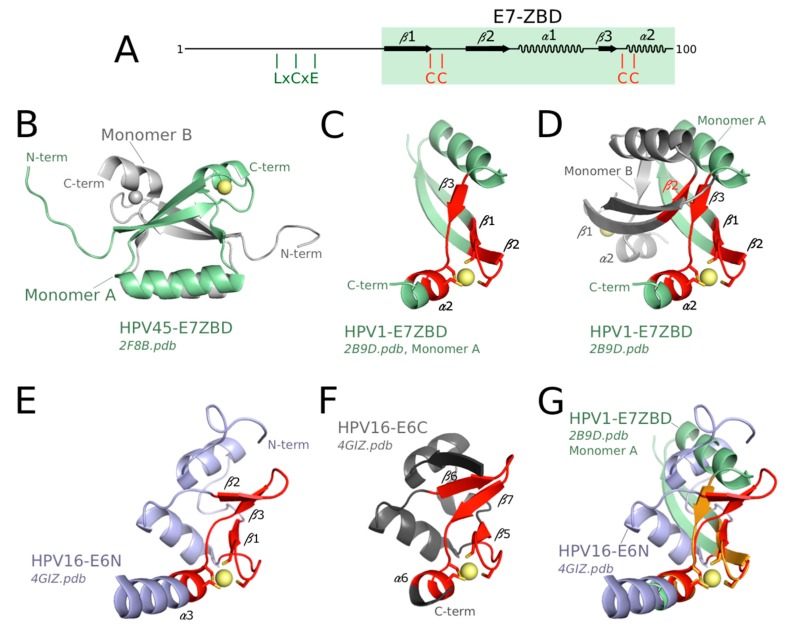
Structure of mammalian papillomavirus (PV) E7 ZBD and comparison to mammalian PV E6N and E6C. (**A**) Schematics of mammalian HPV E7 sequence. Mammalian PV E7 proteins contain an intrinsically unfolded N-terminus bearing a conserved LxCxE sequence (as indicated on the sheme) among other putative interaction motifs, followed by a folded zinc-binding domain (E7-ZBD) shaded in green, with four conserved cysteine (C) residues as indicated; (**B**) Solution structure of HPV45 E7 ZBD homodimer [76]. A comparable structure was also obtained by crystallography for the E7 ZBD of HPV1 E7 [78]; (**C**) Elements of the treble clef motif of E7 ZBD (in red) in context of the E7 ZBD monomer; (**D**) Elements of the treble clef motif of E7 ZBD (in red) in context of the E7 ZBD dimer; (**E**) Elements of the treble clef motif of E6N (in red); (**F**) Elements of the treble clef motif of E6C (in red); (**G**) Superimposition of HPV1 E7-ZBD and HPV16 E6N, enlighting common features of their treble clef motifs, indicated in orange and red color, respectively. Spheres are Zinc (II) atoms.

The structures of the C-terminal zinc-binding domain of E7 (E7 ZBD) from two phylogenetically distant HPVs, namely HPV 1 (low-risk cutaneous species μ-1) and HPV 45 (high-risk mucosal species α-7), have been solved by X-ray crystallography and solution NMR, respectively [76,78]. The backbone structures are quasi-identical for the two species, indicating the high conservation of this fold across mammalian PV species. In contrast to mammalian E6N and E6C, which are both monomeric in solution, the E7 ZBD presents a dimeric conformation (Figure 3B) that is observed both in solution and in the crystal [76,78]. The interface between the two monomers is highly hydrophobic, suggesting that this is an “obligate” dimer, which is highly favored in solution as compared to the monomeric form [102]. In solution, full-length E7 also forms a dimer since characteristic resonances of the dimeric E7 ZBD are preserved in NMR spectra of full-length E7 [76,103,104]. Indeed, the dimeric structure of high-risk HPV16 E7 favors the formation of a ternary complex between E7, pRb and CBP-p300 (CREB-Binding Protein-histone acetyltransferase protein 300) [103].

In addition to being dimeric, the fold of mammalian E7 ZBD is clearly distinct from that of E6N and E6C, with a different secondary structure ordering and topology (Figure 3A, compare to Figure 2A). Nevertheless, both folds probably originate from a very distant common ancestor. This is supported by the conservation of a common core structure comprising the four Zinc-coordinated cysteines and the secondary structure elements bearing these cysteines (Figure 3, compare panel C, E and F). Indeed, this core zinc-binding structural element has been noticed to exist in a variety of proteins including E6 and E7 [105] and was originally described as the “treble clef motif” [106,107]. The treble clef motif contains the following succession of elements: zinc knuckle (i.e., two short β-strands connected by a turn that bear two zinc-binding residues), loop, β-hairpin, and α-helix. Two zinc binding cysteines are contributed by the knuckle, a third cysteine is situated at the junction between the β-hairpin and the α-helix, and the fourth cysteine is contributed by the α-helix. The mammalian E6N and E6C folds contain the four secondary structure elements (Figure 3E,F) and therefore can be classified in this category. The mammalian E7 ZBD monomer contains the zinc knuckle, a loop, a single β-strand, and an α-helix (Figure 3C,D). Two zinc-binding cysteines are contributed by the knuckle, a third cysteine is situated at the junction between the β-strand and the α-helix, and the fourth cysteine is contributed by the α-helix. This organization also fits to the definition of the treble clef, with the difference that the β-hairpin is replaced by a single β-strand (β3, in contact with β2 from opposite monomer, see Figure 3D). Nonetheless, all the secondary structure elements that surround the zinc ion in mammalian E7, E6N, and E6C all superimpose very well (Figure 3G), supporting a common evolutionary origin for these three zinc-binding domains.

Remarkably, the avian E7 ZBD [79] is more similar, by its sequence and overall fold, to the mammalian E6N domain than to the mammalian E7 ZBD. Therefore, avian E7 ZBD and full-length avian E6 are structurally homologous to the mammalian E6N and E6C domains, respectively. This indicates that the mammalian “bidomain” E6 has arisen from the fusion of two ancestral “monodomain” E7 and E6 proteins, that were themselves derived from a single ancestral zinc-binding fold, presenting the common features shared by mammalian E6N and E6C domains. The distinctive dimeric fold of mammalian E7 ZBD must correspond to a very ancient duplicate of the common ancestor of E6N and E6C domain, that has later followed a divergent evolutionary process.

The cysteine-rich and dimeric mammalian E7 ZBD may contribute, depending on purification and/or storage conditions, to the redox-sensitivity and self-association properties of E7, which have been thoroughly analyzed under different aspects by the Prat-Gay group [108,109,110,111]. Interestingly, based on these studies the authors have proposed to use hyperstable E7 oligomers for vaccination against HPV-positive tumors [112]. This demonstrates that basic research focusing on the biophysical properties of HPV oncoprotein folds may also lead to practical applications of direct medical interest.

### 3.5. Conformation of Uncomplexed Mammalian E6 in Solution

**Figure 4:**
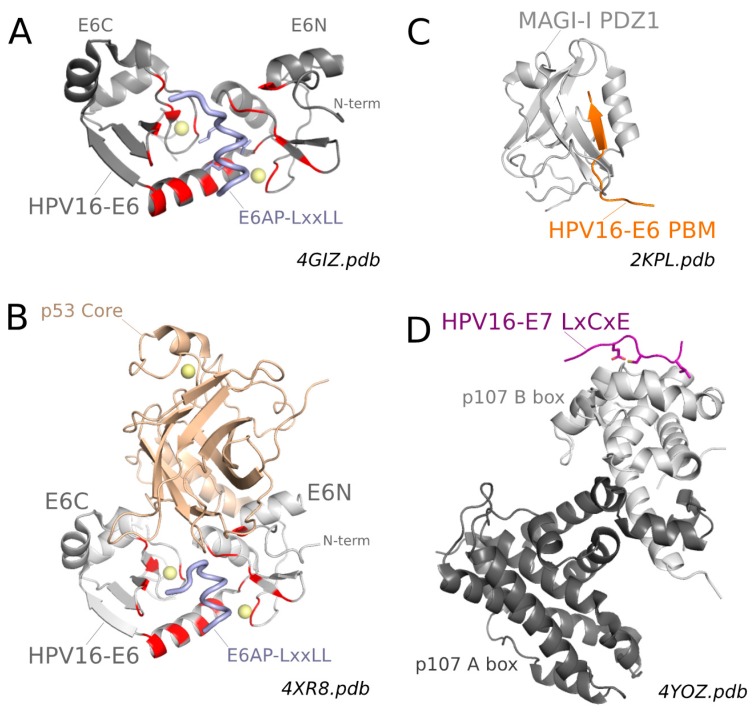
Main structural data on HPV16 E6 and E7 bound to cellular targets. (**A**) Crystal structure of full-length HPV16 E6 bound to the LxxLL motif of E6AP [75] LxxLL binding residues are highlighted in red; (**B**) Crystal structure of HPV16 E6 bound to the LxxLL motif of E6AP and the core domain of p53 [98]; (**C**) Solution structure of MAGI-1 (membrane associated guanylate kinase inverted 1) PDZ 2/6 bound to the C-terminal PDZ-Binding Motif (PBM) of HPV16 E6 [115]. LxxLL binding residues are highlighted in red; (**D**) Crystal structure of p107 pocket domain (composed of A-box and B-box) bound to the LxCxE motif of HPV16 E7 [116]. A comparable crystal structure was previously obtained for pRb pocket domain bound to the LxCxE motif of HPV16 E7 [117].

To date, no crystal structure of uncomplexed full-length mammalian PV E6 could be obtained despite significant efforts. However, the NMR analysis of full-length E6 has provided extended insight on the conformation of free E6 in solution [91]. Most backbone amide signals of the full-length E6 construct could be identified and were found to overlay with signals in its separated E6N and E6C domains. This indicated that the structures of the domains were preserved in the context of the full-length protein. However, most of the resonances of the interdomain linker (residues 75–82) could not be observed, likely due to dynamic processes. NMR measurements of the tumbling correlation time (τc) indicated that the uncomplexed full-length E6 protein behaved as a rigid monomer rather than two independently tumbling domains [91]. This suggests that the interdomain linker may transiently adopt, in free E6 in solution, the helical conformation that is observed in the crystal structure of LxxLL-complexed E6 (Figure 2E, see also Figure 4A). This would represent a typical example of “conformational selection”. Conformational selection is a theoretical framework, which proposes that unliganded proteins already adopt—or “sample”—, in a proportion that varies for each case considered, the conformation that they will adopt in the bound state [113].

While the conformation of free E6 represents an interesting and not fully resolved biophysical issue, it is rather the E6 proteins bound to their host targets that exert biological and pathogenic effects. In addition, full-length HPV E6 proteins appear to be expressed in very low amounts in tumor cells [97,114] so that they may essentially exist in the form of target-bound complexes. These considerations have motivated structural investigation of E6 proteins in complexes with cellular target proteins, as will be described in the next paragraphs.

## 4. Structure and Specificity of E6-Target Complexes

### 4.1. Structure of Full-Length Mammalian E6 Proteins Bound to Target LxxLL Motifs

All mammalian PV E6 proteins recognize peptides presenting the LxxLL consensus sequence interspaced with acidic residues. To date, two X-ray structures of E6 proteins bound to such acidic LxxLL motifs have been solved [75]. First, the structure of a soluble mutant of HPV16 E6, complexed to a construct including the LxxLL motif of the E6AP ubiquitin ligase fused to the C-terminus of bacterial MBP, was solved at a 2.6 Å resolution (Figure 4A). Second, the structure of a triple fusion construct including bacterial MBP, the LxxLL motif of the cellular focal adhesion protein paxillin and the E6 protein of Bovine papillomavirus BPV1 was solved at a 2.3 Å resolution [75].

Despite the low sequence identity (30%) of the two E6-LxxLL complexes, their overall structures were very similar [75]. This strongly supports the notion that LxxLL recognition is a conserved structural property of E6 proteins throughout mammalian papillomaviruses [118], a view corroborated by recent proteomic data on a variety of HPV E6 proteins, that were all found to pull-down LxxLL motif-containing host proteins [31,32,34,36].

As discussed before, the BPV1 and HPV16 E6C domains have a similar fold while the two E6N domains have structurally resolved regions that are essentially superposable but differ in their N-terminal regions, partly due to the fact that the N-terminus of BPV1 E6 was unresolved in the crystal [75]. The LxxLL motif is bound in the form of a helix, which inserts within a pocket formed by the two domains and the linker helix of E6 (Figure 4A). This conserved general mode of recognition relies on the conserved secondary structure topology of the two E6 proteins, and on the conservation of a few key positions that could be identified [75]. Most logically, E6 residues conserved for general recognition were found to interact with either backbone atoms of the LxxLL peptides or side chain atoms of the three invariant Leucine residues.

Comparative examination of the complexes also allowed for identification of subtle amino acid differences at other positions, dubbed “reader” positions, that dictate the discriminative preferences of E6 from HPV16 and BPV1 for the LxxLL motifs of E6AP and Paxillin, respectively [75]. At least two regions of E6 were found to be critical for discriminative “reading” of LxxLL subsets. The C-terminus of the E6 linker helix and the N terminus of E6N domain were suggested to influence the selection of residues at the N or C terminus of the peptide, respectively [75]. Conversely, despite being conserved in HPV16 E6 and BPV1 E6, some reader arginine residues were found to establish contacts with acidic side chains belonging to different turns of the bound helix [75]. In other words, the same reader residue in two different E6 proteins can read a distinct position in the target LxxLL motif. It therefore appears that E6 proteins from different mammalian PVs have undergone sequence evolution mechanisms, which have allowed them to conserve the general capacity to interact with LxxLL motifs while specializing, through subtle variations in their “reader” positions, for the capture of different panels of target proteins bearing variations of the LxxLL motif.

### 4.2. Why Do Apparently Unrelated E6 Target Proteins Contain a Conserved E6-Binding LxxLL Motif? The CBP-P300 Hypothesis

Most mammalian E6 proteins recognize acidic LxxLL motifs [75], yet E6 proteins of distinct PV species recognize different subcategories of these motifs and hence recruit distinct LxxLL-containing host proteins [32,36,75]. The LxxLL motifs targetted by E6 proteins are highly conserved in their respective cellular proteins across mammals and sometimes even across the whole vertebrate lineage. These LxxLL motifs are unlikely to be conserved in diverse host proteins for the sole purpose of binding to viral E6. Rather, they may participate in a common host function, whose perturbation is useful to the viral lifecycle. LxxLL motifs are frequent in transcriptional co-activators, where they mediate crucial protein–protein interactions [119,120]. Remarkably, the best characterized LxxLL-containing E6 targets, i.e., E6AP [121], p53 [122], MAML1 [123], IRF3 [124], paxillin [125] and its close paralog ARA55 (Androgen Receptor Associated protein 55) [126], hADA3 (human Transcriptional Adapter 3) [127] and CCR4-Not complex [128], are all involved in transcriptional activation or co-activation. Moreover, most of them have been shown to interact with the CBP-p300 protein [121,122,123,124,126,127,128], which is itself a direct target of some E6 proteins, such as those of HPV16 and β1 HPVs [36,129]. CBP-p300 is a central transcriptional co-activator with acetyltransferase activity involved in numerous functions, including the host innate antiviral response [124,130]. Therefore perturbation of CBP-P300 activity is a consistent feature of many viruses [131]. Moreover, CBP-p300 is also involved in tumorigenic pathways [132]. Remarkably, CBP-p300 not only contains LxxLL motifs that bind to transcription factors [132,133], but it also recruits LxxLL motifs from co-activator proteins. In particular, the KIX domain of CBP-p300 modulates CBP-p300 activity by binding to acidic LxxLL motifs [134] reminiscent of those preferentially targeted by E6 proteins.

Based on the above-mentioned published observations, it is tempting to speculate that all mammalian E6 proteins share the ability to interfere with CBP-300 activity by directly interacting with CBP-300 or by capturing acidic LxxLL motifs within cellular partners of CBP-p300. This conserved ability to interfere with CBP-p300 activity would not only make sense for mammalian PVs to counteract innate immunity responses, but might also contribute to PV-induced oncogenesis. Indeed, the oncoprotein AML1-ETO, a fusion protein associated with acute myeloid leukemia (AML), competes with CBP-p300 for binding to the acidic LxxLL motif of E-proteins, a family of transcription factors that normally interact via their LxxLL motif with the KIX domain of CBP-p300 [134,135]. In a similar way, E6 oncoproteins might perform part of their tumorigenic action by competitively blocking the access of cellular acidic LxxLL motifs to CBP-p300. Besides, the acidic LxxLL motifs recognized by E6 proteins may also bind and regulate transcriptional activators or co-activators other than CBP-p300, that remain to be identified.

### 4.3. Structure of the Ternary E6/E6AP/p53 Complex

As discussed before, E6 of high-risk mucosal (hrm) HPVs binds to the LxxLL motif of cellular E3 ubiquitin ligase E6AP. The resulting E6/E6AP complex then recruits the p53 anti-apoptotic tumor suppressor protein, provoking its poly-ubiquitination and subsequent degradation by the proteasome [58]. The structural basis of the ternary E6/E6AP/p53 complex formation has been recently elucidated [98]. It was found that binding to a minimal 12-meric LxxLL E6AP-derived peptide was sufficient to render full-length E6 proficient for interaction with p53 [136]. Furthermore, the folded “core” domain (residues 94–292) of p53 was efficiently recruited by the pre-formed E6-LxxLL dimer. The crystal structure of the resulting E6-LxxLL-p53 core trimeric complex (involving bacterial Maltose Binding Protein fused to the LxxLL motif for solubility and crystallizability purposes) was solved at 2.25 Å resolution (Figure 4B). The structure of the E6-LxxLL subunit of the trimeric complex is practically superimposable to that of the E6-LxxLL complex previously solved [75] (Figure 4, compare panel A & B). The p53 core domain is recruited to this complex via a large interface essentially provided by E6. Whereas the presence of the LxxLL motif is required for E6 to recruit p53core, the LxxLL motif does not significantly interact with the p53core in the complex. This indicates that the main role of the LxxLL motif in p53 recruitment is to stabilize E6 in the LxxLL-bound conformation presenting the extended p53-binding interface. In light of our previous discussion of the solution structure of E6, this suggests that, while free E6 may transiently adopt in solution the conformation observed in the crystallized E6-LxxLL complexes, this conformation is not sufficiently sampled to allow for detectable p53 binding in the absence of the LxxLL motif of E6AP.

The p53-binding surface of E6 involves both E6N and E6C domains [98]. It is relatively large (1200 Å^2^), in contrast with the rather weak affinity of the preformed E6-LxxLL complex for p53core (Kd = 22 μM). The weak affinity might be related to the predominance of polar contacts in the E6/p53 interface. In vivo, the affinity of p53 for E6/E6AP is likely to be enhanced by avidity effects, since full-length p53 and E6AP are known to form tetramers [137] and trimers [138], respectively. The E6-binding surface of p53 has not been seen before to be involved in binding to cellular p53 target molecules, including DNA. This suggests that E6 can recruit p53 and drive its degradation even when p53 is complexed to its natural targets.

The p53-binding surface of E6 comprises residues D44 and F47 of the E6N domain, which also mediate E6N dimerization and subsequent self-association of E6 [91]. Therefore, E6 self-association and E6 binding to p53 are two competing processes. However, this does not rule out the possibility that E6 might self-associate when it is not bound to p53.

While the assembly of E6, E6AP and p53 has been revealed, the structural mechanism of the subsequent poly-ubiquitination of p53 remains to be investigated. At least two possible models can be proposed. In one model, the sole formation of the triple complex is sufficient to place the E6AP HECT domain in atomic proximity of the ubiquitinable regions of p53, which thus becomes poly-ubiquitinated without further need of activating E6AP. In a second model, E6 not only induces the formation of the triple complex but also potentializes the ubiquitination activity of E6AP, for instance by inducing an activatory conformational change onto E6AP [139]. To decipher the full mechanism, further structural studies will be required, including the exploration of complexes involving larger parts of the 850 residues composing the E6AP protein. To date, high-resolution structural information on E6AP remains restricted to the N-terminal zinc-binding domain (~80 residues) [140], the central 12-meric LxxLL peptide in complex with E6 [75], and the C-terminal “HECT” domain (~350 residues) bearing the ubiquitin ligase activity [141].

### 4.4. Structural Basis of Hijacking of PDZ Domains and Rb Pocket Domains by High-Risk Mucosal HPVs

As discussed before, viral proteins often evolve mimics of host small linear interaction motifs, allowing them to competitively capture host domains and thereby perturb domain-motif networks carrying out particular biological functions [37,39]. The structural analysis of such interactions is facilitated by the fact that they do not necessarily require production of the full-length viral protein, since they can focus on complexes involving the isolated target domain(s) and short peptides corresponding to the viral motif.

As concerns the conserved PDZ-Binding Motif (PBM) found at the C-terminus of high-risk mucosal E6 proteins, several structures of the isolated E6 PBM have been solved by NMR and crystallography in complex with a variety of human PDZ domains: MAGI-1 PDZ2/6 domain [115,142], DLG1 (Disk Large homolog 1) PDZ2 and PDZ3 domains [78,101,142] and CAL (CFTR-associated ligand) PDZ domain [143]. Expectedly, all these structures demonstrated that, both in solution and in crystals, the viral E6 PBM interacts with the peptide-binding pocket of the PDZ domains following the “canonical binding mode” generally observed for host–host PDZ-PBM complexes (Figure 4C). For the strongest PDZ-E6 PBM complexes, binding affinities were in the micromolar (μM) range [22,71,78,101,115,144,145,146,147]. Subtle variations in binding thermodynamics and/or kinetics could be detected, that depended on the nature and length of amino acid extensions flanking either the N- and C-termini of the PDZ domains or the N-terminus of the PBM used for the interaction assays [101,115,146,147].

Of note, among the structures above mentioned, only the complexes involving PDZ domains from MAGI1 and DLG proteins correspond to interactions likely to be relevant in vivo. The E6-CAL complex [143] is probably physiologically irrelevant as the E6 PBM does not detectably interact with the CAL protein in in vitro assays. Indeed, this E6-CAL complex was investigated with the sole aim of observing at a high-resolution the unfavorable atomic interactions that are responsible for the very low affinity displayed by the E6 PBM for the CAL PDZ domains. Crystallization of this unnatural complex could, however, be achieved, thanks to the very high local concentrations reached during crystal formation. Therefore, the determination of a high-resolution crystal structure of a complex does not always warrant that this complex is relevant in vivo.

The human proteome comprises 266 human PDZ domains distributed over 152 proteins [71,148]. Due to the high degree of conservation of their structure and peptide-binding mode, PDZ domains can be relatively promiscuous, i.e., several PDZ domains may compete for binding to the same PBM-containing proteins. This illustrates the interest of recent studies [70,71] (see paragraph 2.5), that aimed to address in a quantitative way the preferences of E6 proteins towards the entire set of host PDZ domain-containing proteins. Such unbiased proteome-wide quantitative studies, combined with high-resolution structural data, will help understanding how subtle sequence variations of E6 proteins rule their preferences for different pools of target host proteins, thereby impacting the viral and pathogenic phenotypes.

As concerns the conserved LxCxE motif found in the N-terminal region of high-risk mucosal E7 proteins, two crystal structures of this isolated motif have been solved in complex with the Pocket domains of two different members of the Rb protein family: pRb [117] and p107 [116] (Figure 4D). These structures demonstrated that the viral E7 LxCxE interacts with the peptide binding groove of the pocket domains, in the same way as the equivalent LxCxE motifs from host proteins [116,117].

## 5. Exploitation of High-Resolution Structural Data for In Vivo Inhibition of E6 Oncogenic Activity

Despite their very recent release, the high-resolution structural data available on E6-target complexes have already started to be utilized for the exploration of potential therapeutic applications.

As discussed above, all high-risk mucosal E6 oncoproteins bind to PDZ domains and LxxLL motifs; both of these activities are crucial for HPV-induced oncogenesis; and high-resolution data are available on both E6-PDZ and E6-LxxLL complexes. This inspired two distinct research groups to design chimeric PDZ-LxxLL [22] and LxxLL-PDZ [149] fusion proteins. Both types of constructs were shown to act as strong bivalent E6 ligands displaying a nanomolar affinity. Furthermore, the PDZ-LxxLL chimera was shown to bind strongly and specifically to E6 proteins of all high-risk mucosal HPV types, and to provoke apoptotic death of HPV-positive cells derived from cervical tumors [22].

In another study, pep11**, a peptide displaying potent pro-apoptotic effects in HPV16-positive cells [24], has been probed by NMR for its specific interaction with full-length HPV16 E6 in solution [23]. The interaction was shown to be in the nanomolar affinity range, and to induce time-dependent aggregation and subsequent precipitation of E6. Prior knowledge of most backbone frequencies of HPV16 E6 [91] allowed for the mapping of the pep11**-binding surface on HPV16 E6. This surface was found to overlap with the E6AP-binding surface of E6, thereby providing a strong structural basis to the likely hypothesis that the pro-apoptotic effects of pep11** are mediated, at least in part, by its specific binding to HPV16 E6.

Finally, the structural data on E6/E6AP complexes have been utilized for in silico docking of small molecule E6 ligands displaying E6 inhibitory activity in the micromolar affinity range. It should be noted however, that the docking onto E6 3D structure did not serve the design of the small molecules, as it was only performed *a posteriori* to verify that the molecules characterized in this work could potentially be accommodated within the E6 pocket [28,30].

## 6. Conclusions

High-resolution structural analysis of the multifunctional E6 and E7 oncoproteins had been awaited for many years after their discovery. In the last decade, progress in production and solubilization of these proteins paved the way to breakthrough advances. The structures of zinc-binding domains of several E6 and E7 proteins have been solved by NMR and crystallography, revealing two zinc-binding folds probably derived from a common ancestor. Next, combined structural and biophysical data have depicted the strategies employed by E6 and E7 oncoproteins to hijack host domain-motif interaction networks. Several X-ray and NMR structures have described the mode of binding of short linear interaction motifs from both E6 and E7 to target host domains (PDZ and pocket domains, respectively). In parallel, quantitative proteomics approaches have been developed to identify and rank among the entire complement of human PDZ domains, those which bind best to each HPV E6 oncoprotein analyzed. Furthermore, X-ray structures of two E6-LxxLL complexes have described commonalities and differences in the recognition of host LxxLL motifs, a conserved property of most mammalian E6 proteins. Very recently, a trimer composed of HPV16 E6, the LxxLL motif of E6AP, and core domain of p53 has been solved by X-ray crystallography, depicting the mode of assembly of a viral p53 degradation complex.

Nonetheless, there are still exciting avenues to explore concerning the biophysical and structural characterization of HPV oncoprotein activities. It will be interesting to further investigate the molecular basis of papillomaviral motif/domain hijacking strategies by combining quantitative proteomic analysis of the LxxLL interactomes of diverse human or mammalian PV species, with the resolution of additional E6-LxxLL structures of particular interest, such as α-HPV E6/IRF3, β-HPV E6/MAML1 and β-HPV E6/CBP-p300. In these complexes, it will be important to extend the size of the host protein constructs involved by including, whenever possible, the full-length cellular proteins, rather than their sole LxxLL motifs. A particularly challenging aim will be to visualize at high resolution the p53 degradation complex including full-length E6 protein, full-length E6AP ligase (850 amino acids, possibly trimeric) and full-length p53 tetramer (4 × 393 amino acids). Last but not least, a research with high potential impact on human health will consist of exploiting the novel high-resolution structures of HPV oncoprotein–host protein complexes for the design and optimization of small molecule inhibitors of the pathogenic activities of HPVs.
